# Semantic Agent-Based Service Middleware and Simulation for Smart Cities

**DOI:** 10.3390/s16122200

**Published:** 2016-12-21

**Authors:** Ming Liu, Yang Xu, Haixiao Hu, Abdul-Wahid Mohammed

**Affiliations:** 1 School of Computer Science and Engineering, University of Electronic Science and Technology of China, Chengdu 611731, China; mingliu.uestc@gmail.com (M.L.); 201511060118@std.uestc.edu.cn (H.H.); 2 School of Engineering, University for Development Studies, Tamale 00233, Ghana; abdulwahid.mohammed@uds.edu.gh

**Keywords:** smart city, agent-based middleware, semantic service, M2M

## Abstract

With the development of Machine-to-Machine (M2M) technology, a variety of embedded and mobile devices is integrated to interact via the platform of the Internet of Things, especially in the domain of smart cities. One of the primary challenges is that selecting the appropriate services or service combination for upper layer applications is hard, which is due to the absence of a unified semantical service description pattern, as well as the service selection mechanism. In this paper, we define a semantic service representation model from four key properties: Capability (C), Deployment (D), Resource (R) and IOData (IO). Based on this model, an agent-based middleware is built to support semantic service enablement. In this middleware, we present an efficient semantic service discovery and matching approach for a service combination process, which calculates the semantic similarity between services, and a heuristic algorithm to search the service candidates for a specific service request. Based on this design, we propose a simulation of virtual urban fire fighting, and the experimental results manifest the feasibility and efficiency of our design.

## 1. Introduction

Machine-to-Machine (M2M) technology offers powerful support for integrating various devices, equipment and units, gives rise to new synergistic services for the smart city and goes beyond the services that can be provided by an isolated embedded system [1]. Recently, the leading standardization organization oneM2M [2] presented a normalized semantic M2M supporting framework, which integrates products and design standards from various manufacturers, shielding the heterogeneous differences in order to provide users with better service-oriented support.

Any standardization work has to go through a long stage of development and would be influenced by many factors, especially the constant emergence of new technologies. The current barrier is that every application has respective domain characteristics, strong independence, heterogeneity and difficulties with respect to reusability [3]. Albeit that some works provide registration-based service discovery and response mechanisms, the resources are scattered in every corner of the system, and it is still difficult to resolve the heterogeneity and loose coupling. As a consequence, a new middleware design pattern should be added to the existing M2M applications to support the general semantic service processing and scheduling of tremendous services within the system. This is the main motivation of the work proposed here. As the principal component in this paper, the authors introduce an agent-based middleware design for semantic service support and enablement in detail and discuss its performance in urban fire response.

The rest of the paper is organized as follows: Section 2 overviews and analyzes the related work on agent-based middleware design and some current M2M platforms in line with the European Telecommunication Standards Institute (ETSI) standard. By analyzing the deficiencies of the current work, it expounds the necessity of semantic-oriented middleware design. Next, the authors propose a novel middleware design pattern and give details in Section 3. The service description and ontology definition are presented in Section 4. Services’ discovery, matching, combination and scheduling mechanism are detailed in Section 5. The evaluation of the proposed design and simulation results are presented in Section 6. Finally, the conclusion and future work are given in Section 7.

## 2. State of the Art

Recently, M2M technology has received significant attention from both industry and academia. As an emerging paradigm, M2M can manage billions of devices, resources, data and actuators and gives strong support to the new smart city era [4]. Curwen, P. et al. [5] estimates that in the next two decades, there will be more than 20 billion devices providing diverse services and bridging different domains with more connections and interactions based on Radio Frequency Identification (RFID), Wireless Sensor Network (WSN) and other entities. In the traditional service domain, services are typically defined by a set of Hypertext Transfer Protocol (HTTP) request messages along with a definition of response messages and follow such principles as the Simple Object Access Protocol (SOAP), Representational State Transfer (REST) [6] and Extensible Markup Language-Remote Procedure Call (XML-RPC) [7]. The primary objective of SOA is to facilitate service integration between independent entities or organizations by using a set of service publication and discovery facilities. To facilitate diversification and a convenient service support, some approaches, such as the Web Service Description Language (WSDL), Universal Description Discovery and Integration (UDDI), as well as Web Ontology Language (OWL), Web Service Semantics (WSDL-S) [8], Semantic Annotations for WSDL and XML Schema (SAWSDL) [9], have been specified for helping the publication component to better describe the services and the discovery component to more easily identify the right content/service matching the user’s requests, context and preferences.

Middleware provides an encapsulated software function layer between the application layer and the network communications layers and which facilitates and coordinates the integration of heterogeneous computation and communication devices, supporting the interoperability within the diverse applications and domains [10]. Some research proposed the agent-based approach to middleware design, where an agent can proxy a specific function or device and cooperate with other agents to proactively gather data and update the state of the system. Typical agent-based middleware solutions are as follows: ActorNet [11] is designed to improve the interoperability in multitasking execution in resource-constrained Wireless Sensor Network (WSN) applications. Agilla [12] provides self-adaptive mobile agents to proxy sensors within the WSNs. Each agent is designed to be event driven and maintains a tuple space to enable the resource discovery. Some other similar middleware are Ubiware [13], TinyMAPS [14], etc. A drawback of these middlewares came from the design mechanism used; they do not address the heterogeneity of M2M, and most middlewares cater to WSNs or lightweight mobile devices. Moreover, these middlewares cannot support a complex application possible in more resource-rich devices and lack support for semantic and syntactical interoperability. Furthermore, extensibility and operation management pose a challenge because of the low level of resource abstraction. Some researches also proposed a semantic-oriented middleware design, and PSWare [15], Hydra [16], UbiROAD [17] and SENSEI [18] are typical examples. They all provide a lightweight semantic layer that separates the application from the underlying hardware, operating system and network infrastructure. Moreover, some agent-based middlewares were proposed in [19,20,21] that integrated a context-aware model and a resource-service layer. These middlewares provide a virtual function entity working between the application and communication service layers in M2M applications.

The European Telecommunication Standards Institute (ETSI) provides a resource-oriented M2M standard with a generic set of capabilities for M2M services [22]. It defines the service capability layer as a provider shared between different applications, M2M devices and a subnetwork, which is shared by different applications. Some current M2M platforms in line with the ETSI standard are in Table 1. The corresponding characteristic of a platform is marked with “△”. The resource sharing feature indicates that these platforms can share the intrasystem resources with other platforms, which have applied the same resource definition schema and protocol. The data analysis feature indicates that these platforms can provide the content analysis and representation of the underlying data, not just the data package transport [23], and the final column briefly presents some characteristics of the platform. As a primary member, ETSI together with six other international organizations jointly established the oneM2M organization [24]. The oneM2M inherited the existing design of the ETSI standard, proposed a general architecture specification for an end-to-end M2M system and is compatible with many existing M2M designs. It introduces a novel semantic supporting functional model (Layer) in oneM2M Technical Specification (TS)-0007 [24]. However, the current work is more focused on specifying the interfaces used to interact with external entities and only gives a brief functional description, which follows the popular REST operation and interfaces; it is still difficult to cater to a more sophisticated demand. The oneM2M wants to promote an easy creation of interworking proxy functions, and the resources can be built according to the information model; however, these resources should contain a special attribute that contains a link to the XML Schema Definition (XSD) file of the information model [24], but such work is still underway.

Although partial existing middlewares can support the abstraction by its nature, most of the current work still lacks the support of the semantic service enablement and the resource constraint; in addition, their predefined and deterministic composition mechanisms will not scale well in dynamic M2M applications. In addition, most of the existing work are WSN-centric, and their scale is limited to WSNs, which is typically in the range of thousands; there is no standard ontology design principle for scalable, complex and dynamic M2M environments. Consequently, an agent-based, scalable and semantic-oriented middleware is required, and in this paper, we are more focused on a smart cities-oriented semantic service framework design, operation and support.

## 3. Design of the Agent-Based Middleware

### 3.1. The Basic Model

**Figure 1:**
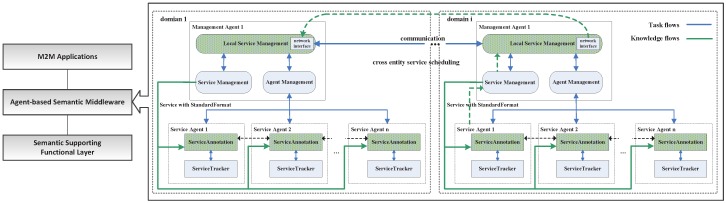
Middleware architecture and cross entity service scheduling.

An abstract M2M middleware system can be modeled as shown in Figure 1; it can be built with three layers: the M2M applications layer, which provides an interface with various M2M applications; the agent-based semantic middleware layer, which performs the main functionalities for service creation and enablement for M2M data and devices; the semantic supporting functional layer provides semantic abstraction and connections with a device and/or accessing M2M data; it inherited the main features of the semantics support functional model, which is defined in oneM2M technical specification TS-0007 [24]. The middleware adopted the characteristics of autonomous, proactive and social capabilities of the multi-agent, in distributed service scheduling and management. It keeps the semantic benefits in the former approaches [15,16,17,18] and has been extended from our previous multi-agent platform design [25]. When the upper-level application needs service support, the middleware generates a cross entity service scheduling, as shown with the green dotted line.

There are two kinds of agents in the middleware: the Management Agent (MA), which manages the service agents; and service execution: request response (local or cross domain), annotation, matching, combination, etc. Each MA is in charge of a service domain, which is composed of several tightly coupled entities, such as hundreds of sensors or e-health equipment in a local area. The Service Agent (SA) represents and maintains an entity’s services (device, resource or data source, etc.) in a specific service domain. In this middleware, M2M entities are abstracted into different service agents, which can be mutually scheduled between the management agents. The MA is in charge of the request service scheduling and selecting the best service or a service combination. The SA is in charge of maintaining the proxy service, complete registration of the service and publishing service to the management agent.

#### 3.1.1. Management Agent

The main goal of an MA is executing the services requested by the upper applications and returning the filtered services. The specific functions of this agent include: managing the SAs and service discovery, matching and combination. The demand for services may be a combination of several services within the current system. Therefore, depending on the complexity of finding the right service, the main MA’s goal would usually be separated into several sub-goals. The important beliefs for an MA are: the service request description, which contains the service requirements, constraints and other restrictions of the services.

**Figure 2:**
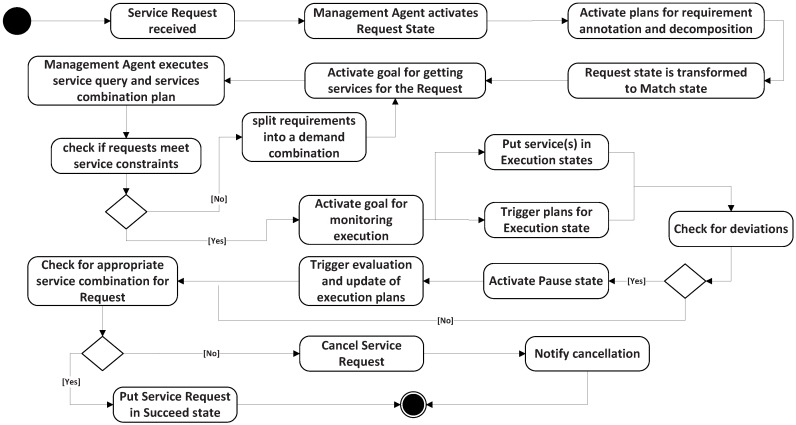
Management agent execution states diagram.

The execution states and implementation of the MAs are listed in Table 2. When a service request is received, the MA activates one or several sub-goals and triggers the execution of the plan to find services to meet the demand. These plans model how the MA behaves for each situation. For instance, when a new service execution is in the request state, MA activates the goal for getting services for the request and executes the plans’ Constraints (Cons.) annotation of the service Requirement (Req.) and its decomposition. An appropriate decomposition changes the service state to match. In match, the MA first executes the query plan. If it cannot return the satisfied constraint services, the requirement will be split into a demand combination. In execution, the MA activates its sub-goal to monitor the service scheduling of the managed SAs. It triggers two corresponding plans: scheduling and Evaluate (Eval.) matching. Scheduling is searching the candidate services in the local and the MA’s neighbors. Eval. matching is evaluating whether the requirement is fulfilled. If all of the constraints have been satisfied, MA will change the state to succeed. If any deviation exists, MA activates the pause status, which triggers the evaluation and updates the plans, until it returns an appropriate service combination (status succeed) or a failure. A service request reaches a cancel status when the service fails to meet the requirement. In this case, the MA falls to recover from the status of execution, and this will be notified as cancel. The execution states diagram is shown in Figure 2.

MA can only manage its local services and store it neighbors’ services, but cross domain service scheduling needs to query the other MAs. The query is based on its observation of other MAs, so it can be a directional query rather than broadcast queries. In addition, as the system expands, each MA may only know its neighbors’ services rather than the whole system (global synchronization states in a large-scale system; the cost is too high and unnecessary). Furthermore, each service within the system is constantly changing (affected by its entity state), and the update is not reported to each neighbor synchronized; it is often based on the query. Therefore, establishing a decision model to support cross domain service scheduling is needed; the details are in Section 3.2.

#### 3.1.2. Service Agent

The main goal of an SA is to maintain its proxy services. The specific functions include: service registration; service publication; and maintaining the semantic description of a service. For achieving this, the services’ semantic information should be provided. These are the semantic properties and capabilities described by the agent, which are related to the current status of the service. In addition to the status, a set of sub-goals and plans has been defined to model the SA’s behavior. These details can be found in Table 3.

**Figure 3:**
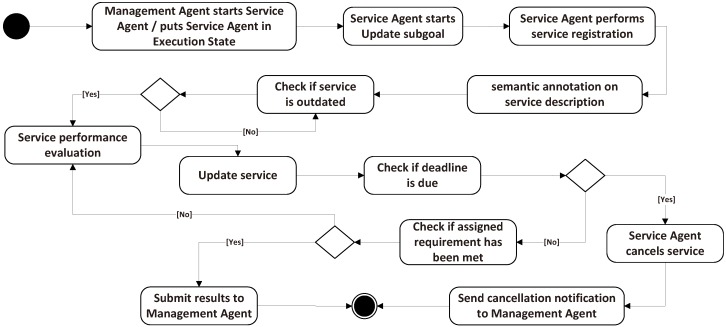
Service agent execution states diagram.

SA has three states: maintain, pause and cancel; and three corresponding sub-goals. Every time a sub-goal is activated, the upper MA will execute a sub-goal to monitor the scheduling of this SA, as defined in the execution state. In the sub-goal of update, SA achieves its local service registration, semantic annotations and updates of its proxy entity periodically. When a service request arrives, MA executes scheduling based on the existing service registration. The sub-goal recover pause is executed for the performance evaluation (Eval.) calculation based on the outdated check. If the service is outdated, SA evaluates the service to decide whether to update or renew the service. The execution states diagram is shown in Figure 3.

In the cancel state, SA prepares to reach service failures. It checks the status and the deadline of a service. If the deadline is due, the SA cancels the service and sends a notification to the MA whose services were scheduled, but failed; the MA treats these notifications according to the plans explained in Section 3.1.1. Once the service is active, SA annotates the service description and registers it to the MA to support the service discovery and scheduling. Furthermore, in the scheduling process, if the current service cannot meet the requirement, the SA submits a message to the MA to inform it of the mismatch. The scheduling process is explained in detail in Section 3.3.

### 3.2. Management Agent Decision Model

Each management agent in the middleware abides by a Belief-Desire-Intention (BDI) model [26]; a set of beliefs, desires and intentions are defined to model the behavior of the agent. At runtime, the BDI engine monitors the agent’s belief, which is the state about its managed SAs and neighbors’ SAs, together with a probability distribution. The desire is represented by the agent’s goal, which is aimed at maximizing its utility reward. The intentions are agent’s behaviors to realize the goal, which seeks to maximize the expected utility. The decision model can be modeled as the following four basic elements: perception, desire, belief and intentions.

- Perception: Agent perception constitutes its local knowledge $k^{t-1}$ with its local service states. Formally, let $S_{i}=\left\{s_{1},s_{2},\ldots ,s_{n}\right\}$ denote the set of local services of $MA_{i}$. Then, $MA_{i}$’s perception is defined as the function $J_{i}:\Omega \to Q_{i}$. If the current perception is *ω*, then $MA_{i}$ knows only that the state of the system belongs to the set $J_{i}\left(t\right)=<J_{i}\left(\omega^{0}\right),J_{i}\left(\omega^{1}\right),\ldots ,J_{i}\left(\omega^{t}\right)>$, and $J_{i}\left(\omega^{t}\right)$ denotes the perception of $MA_{i}$’s in time *t*.
- Desire: $MA_{i}$’s desire $P_{i}^{t}=\left\{p_{1},p_{2},\ldots ,p_{k}\right\}$ can be described by its aspiration level $\alpha_{i}$; it is defined by the task requirement. Utility function $u_{i}$ denotes the rewards of selecting services, which is based on its perceptions. Desire is aimed at finding the max reward in the current local services: $\arg\max u_{i}\left(S_{i}^{*}|P_{i}^{t^{\prime }}\right)\geq \alpha_{i}$ and $S_{i}^{*}\in S_{i}$, $P_{i}^{t^{\prime }}\in P_{i}^{t}$.
- Belief: At time *t*, the whole system’s service states are denoted as ${\bar{S}}^{t}$, $MA_{i}$’s belief $bel_{i}^{t}$ is a distribution on ${\bar{S}}^{t}$ and $J_{i}\left(t\right)$. $MA_{i}$’s knowledge can be denoted as $k^{t}=\left\{bel_{i}^{t},J_{i}\left(t\right)|k^{t-1}\right\}$.
- Intentions: The agent’s intention can be denoted as an action function of $bel_{i}^{t}\times u_{i}\left(i|bel_{i}^{t},k^{t-1}\right)\to i$, which means the action *i* is based on its perception and belief; $i^{*}$ is regarded as an optimum action.

**Figure 4:**
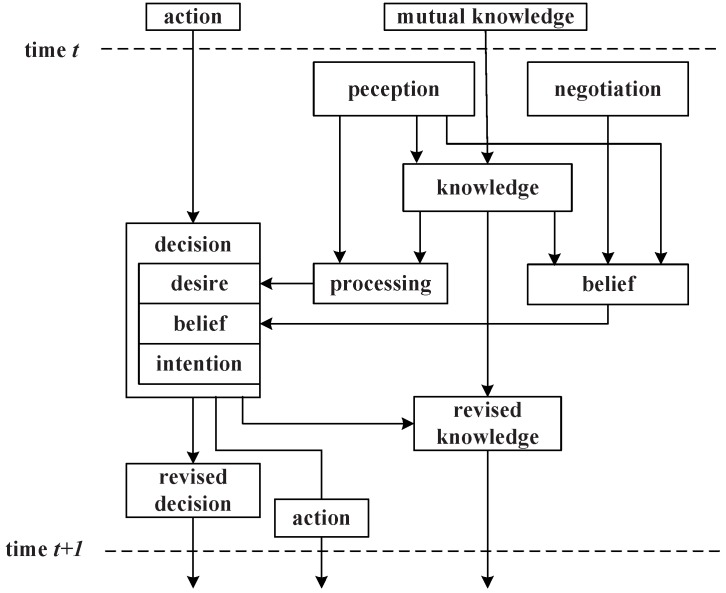
Decision processing in the middleware.

According to the above definitions, the agent activates the decision model by executing the plan and actions to reach its goals. The decision processing is shown in Figure 4.

- From perception to $k^{t}$: $MA_{i}$ gets its local perception $J_{i}\left(\omega^{t}\right)$; it knows that the true global state belongs to the set $J_{i}^{-1}\left(t\right)$. Hence, this transformation consists of determining and removing the inconsistent global states at time *t* with the local observation $J_{i}\left(\omega^{t}\right)$ from $k^{t-1}$.
- From perception to belief: $MA_{i}$ knows the services of its neighbors, and it can deduce roughly what services they can provide. If a service request comes, $MA_{i}$ checks its service registration list and finds the probable services maintained by its neighbors $MA_{j}$ and $MA_{k}$, then sends the query to the two agents; when receiving feedback, $MA_{i}$ knows the services’ current states; $MA_{i}$ can get its revised knowledge $k^{t}$ and make the service selection.
- From decision to revised decision: If $i\times P_{i}^{t^{\prime }}$→$\arg\max u_{i}\left(S_{i}^{*}\right)$, that is the action *i* is consistent with desire, then $i=i^{*}$, and $MA_{i}$ will remove these impossible (inconsistent) desires from the set $P_{i}^{t}$. Meanwhile, $MA_{i}$ may deduce what should be its neighbors’ ground state at time *t*.

### 3.3. Distributed Service Scheduling

**Figure 5:**
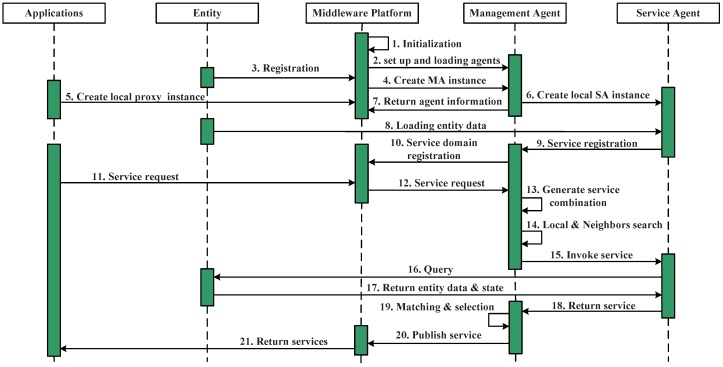
Service scheduling process.

In the distributed service scheduling approach, the service scheduling for a particular request is decided between the management agent and a set of service agents using the aforementioned decision model. Figure 5 shows the workflow during the service scheduling process. Steps 1–9 achieved the agents instantiation and service registration procedures.

When the middleware receives a service request from the upper layer applications, it sends the request to a particular service domain management agent, the management agent selection is based on the registration in the middleware. Based on the service registration and annotation, the management agent builds a query for selecting a candidate service agent in Steps 13–17 (after obtaining the services’ combination table). In this process, the management agent checks its local service list and its neighbors’ service list and finds the candidate service agents, whether they are in the local are; in this case, the first step of service invoking is finding the candidate service agents that fulfill the requirements instead of directly looking for the services. Once the service agent has been selected, the service agent initiates the query process to update its proxy service, when the new data and state are returned, The management agent initiates a matching process to evaluate all of the received candidate services and selects the best ones for the current needs while rejecting the others, in Steps 15–17. Then, it returns the service or the services’ combination to the application (Step 20).

## 4. Enriching the Functionality Building of the Service Ontology

In this section, we give the service ontology design and demonstrate the constraint annotation process, the constraint of which is obtained from the upper application’s requirement.

### 4.1. General Service Design Pattern

In most current work, semantic web services are concentrated on the service’s input, output, condition and enablement, e.g., Ontology Web Language-Service (OWL-S) ontology, Semantic Annotations for WSDL and XML Schema (SAWSDL), Web Service Modeling Ontology (WSMO) and Semantic Web Services Ontology (SWSO). In addition, the W3C Semantic Sensor Network Incubator group (the SSN-XG) introduced a Stimulus-Sensor-Observation (SSO) design pattern to model sensor data in the Semantic Sensor Network (SSN) ontology [27]. Hence, we followed the bottom-up data flow and the REST operation style and extended the aforementioned design pattern to achieve a more generic framework.

**Figure 6:**
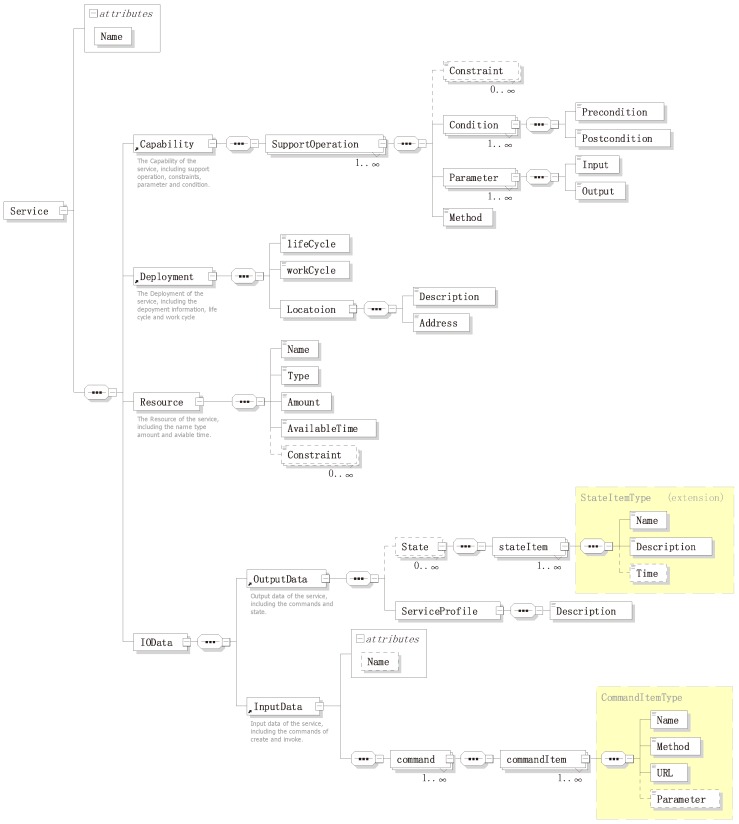
Service XML schemas definition.

For any service, it should have some basic characteristics, such as: name, capability, location, resources (can be null) and its input/output. The ontology takes a liberally-inclusive view of what a service is: any characteristics that it has and allowing such services to be described at any level of detail; for example, allowing services to be seen simply as objects that play the role of ID, as well as allowing services to be described in terms of their components and method of operation. Defining an ontology by the basic characteristics can increase the modularity and reusability of an ontology. Thus, the ontology should enable the service aspects of the use cases, without needing to fulfill all of the modeling requirements. As previously mentioned, for a more convenient service scheduling, in this paper, a four-component-based service design pattern was proposed, describing them in a detailed design model shown in Figure 6. This design model acts as a framework to semantically annotate through building the service schema and the service ontology.

#### 4.1.1. Input and Output Data

**Figure 7:**
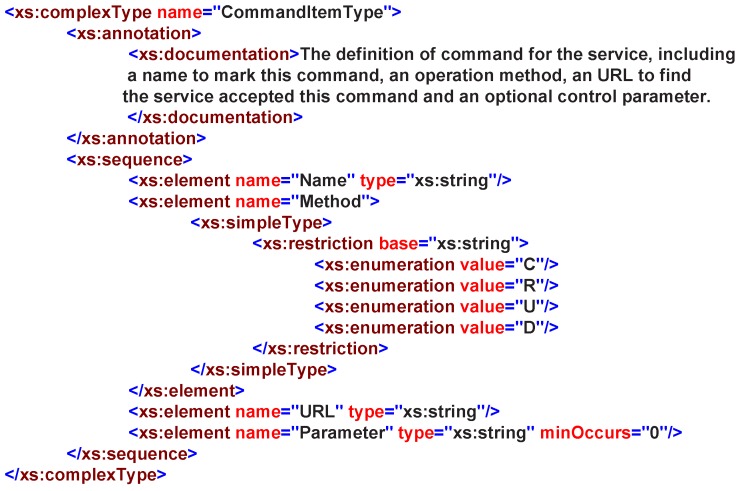
CommandItemType in service XML schemas definition.

The InputData property describes the commands that shows how to invoke the service. The OutputData property describes the semantic information of the service, which includes the service state information (it is dynamically changed) and the service profile. In the service profile, it described the service basic information and the encapsulation of underlying data, which need to be understood and parsed by the scheduler. According to the IOData property, the SA can get all-around information about the service and the transmitting data for its proxy entity. All information will be used to build the service and provide semantic information to support semantic service enablement. Consequently, two complex data types were defined in the IOData property, CommandItemType and StateItemType. This follows the versatile REST operation methods: create(C), retrieve(R), update(U) and delete(D) are involved and supported. A CommandItem contains four basic properties as in Figure 7:

- Name: this indicates the name of the command, and it is helpful in linking this command to the domain knowledge.
- Method: this describes what operation is performed on the service.
- URL: this formally describes the command as a service and points to it.
- Parameter: this describes the parameters required by the command in key-value pairs.

**Figure 8:**
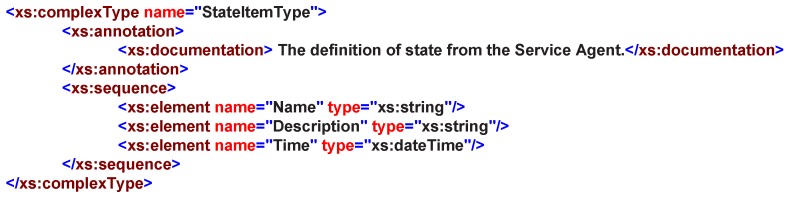
StateItemType in service XML schemas definition.

In web service domain, the definition and description of a service state is often complex and inconsistent; each system has its own service state-defined formats. Hence, towards a semantic-oriented service design pattern, a general state can be described by its Name, Description and Time, as shown in Figure 8. Moreover, more information should be allowed to be annotated as a instance.

#### 4.1.2. Capability, Deployment and Resource

**Figure 9:**
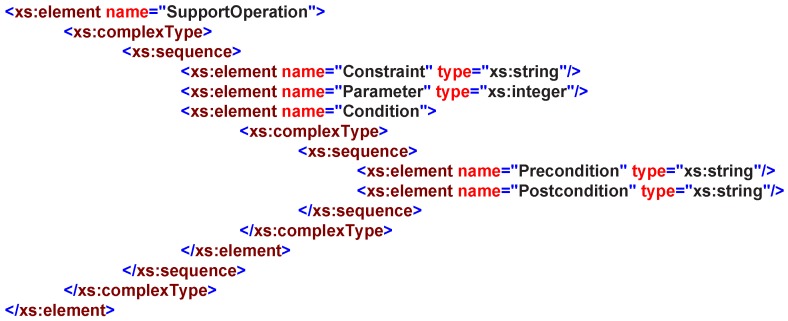
SupportOperation in service XML schemas definition.

The capability property illustrates the proxy entity’s specific features, namely SupportOperation, as shown in Figure 9; e.g., the air condition’s capability can be described as indoor temperature changes; the sensor’s capability can be described as its environment measurement, etc. The apparent characteristic of modeling methods nowadays is that models only apply to specific vertical domains, resulting in poor compatibility and completeness. In this service design pattern, the capability is designed to support the operations that the service can provide, including operations Constraints, Condition, Parameter and Method. This design pattern also simplifies the capability searching process in finding an appropriate service and provides the semantic capability information to better support the service discovery.

**Figure 10:**
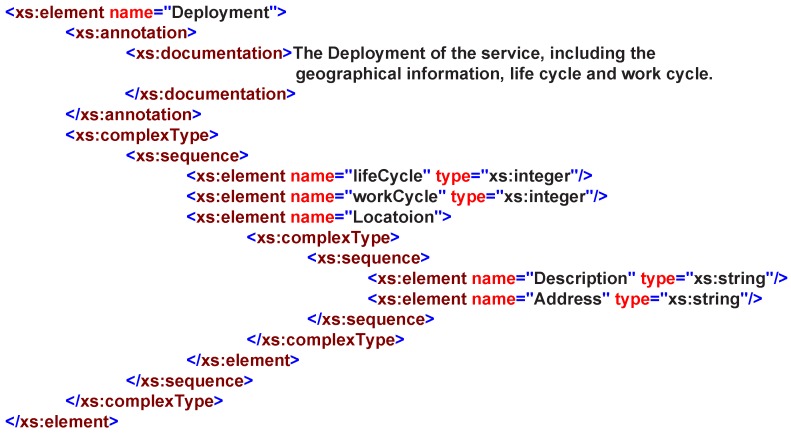
Depolyment in service XML schemas definition.

The deployment property describes where and when a service can be invoked. A management agent shares its maintained services with its neighbor agents and accessed by other agents based on agent’s deployment description. Nowadays, affiliation is considered the most common way to describe the service deployment information. In one way, the deployment is a property that reflects all features of the service agent and the service provider, as shown in Figure 10. In another way, the deployment affiliation relationship reflects the service distribution and their invoking location. The deployment property of services can be described from three elements: lifeCycle, workCycle and Description.

**Figure 11:**
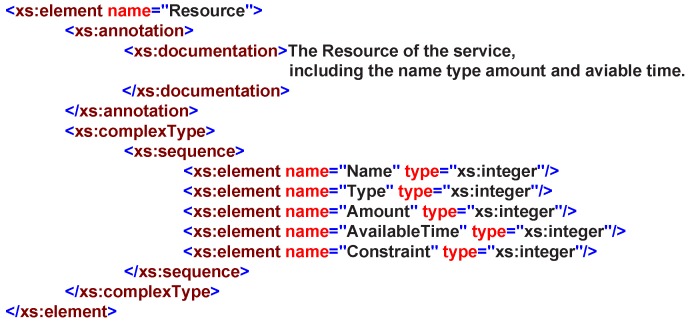
Resource in service XML schemas definition.

A service resource is typically represented as the service can access the underlying functionality elements, such as: entities, data, etc. In RESTful operation methods, a service is usually treated as a resource; therefore, it is easy to achieve the resource-oriented service invoking and accessing other resources that exist within the system. Therefore, in our design, we provide five basic elements to describe the resource: Name, Type, Amount, AvailableTime and Constraint, as shown in Figure 11.

### 4.2. Service Description Ontology Building

Even though the semantic supporting functional layer provides the basic analysis and query function to support the semantic enablement, upper applications still cannot directly access the appropriate services with different requirements. Therefore, we designed a general service ontology based on the XSD design in Section 4.1.

**Figure 12:**
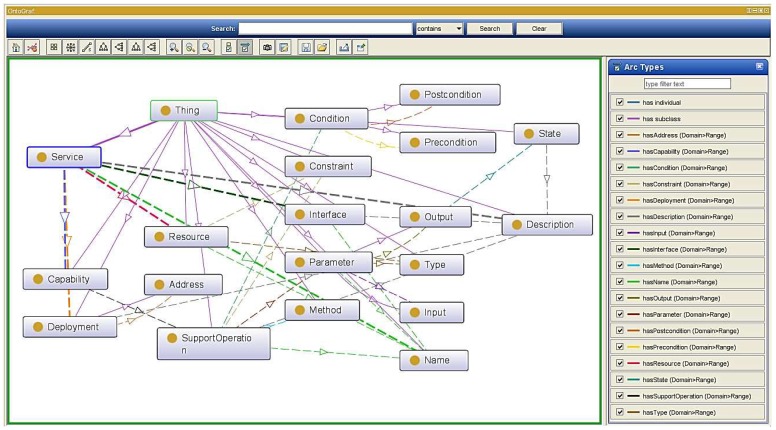
General service description ontology.

Figure 12 illustrates our general service ontology where the oval box indicates the service abstraction describing a concept, solid arrows indicate an object’s attribute definition and dash dotted arrows indicate inheritance relationships between concepts. It defines a service with multiple interfaces, and each interface definition includes multiple actions. Actions are defined as independent functional descriptions in the description of the SupportOperation, which plays a very important role. In addition, to define the parameters of each parameter name, the parameter types are also defined as shown in the XSD definition file indicated earlier. We can also have semantic annotation properties for each parameter element defined in the system. For each type, simple type and complex type, we can also define semantic annotation.

As previously mentioned, the underlayer heterogeneous data can be transformed into a structured XML. These data files are in compliance with a unified data structure and can support the interaction with upper-level applications. However, these data when contacted by an M2M application still cannot be understood, as well as what data are provided by the entity and what these data mean. As a consequence, it will be difficult to meet overwhelmingly complex application requirements and also difficult to filter out the required data from the multiple services. Therefore, we designed the compatible generic service description ontology to fill this gap.

Each service is associated with a service domain, which has an interface and potentially many implementations. Considering that an ontology can formally describe semantic information and for completeness and consistency of our semantic model, we construct the service ontology based on the aforementioned design model and four properties with range classes: output, capability, deployment and resource are the major elements. These classes have their respective subclasses, as well. Besides, the correspondences between XSD and the ontology enable the standardized, structured data to instantiate the service ontology. Thus, the semantical annotation of information will be accomplished after generating a service ontological data file. In particular, current web service designs usually regard the service Quality of Service (QoS) factor. Considering the QoS requirement in real semantic service applications, the service quality of the service data extracted from the underlying entity can only be judged by the service scheduler or the consumer. Besides, there are many services in the system, and services that provide the same functionality will co-exist with a number. Hence, in our design, the service ontology does not include QoS, and it acquiesces the aggregated services to the middleware, which has consistent default qualities.

## 5. Service Discovery and Combination

We will present in this section the details about the service discovery, matching and combination processing. The unitary requirement can be obtained by service discovery and matching, but the complex requirements need to be decomposed, acquire the service combination and then perform a service discovery and matching. Based on the aforementioned middleware design, a middleware-based distributed service scheduling problem with multiple entities can be described as a tuple <$R_{i},\Lambda_{i},I,\Omega ,S,V$>, where:

- $R_{i}=\left\{r_{1},r_{2},\ldots r_{n}\right\}$ denote the current service combination request and $r_{i}$ is a segmented minimum requirement.
- $\Lambda_{i}=\left\{AM_{1},AM_{2},\ldots AM_{K};\times_{K}\left[AS_{1},AS_{2},\ldots AS_{N}\right]\right\}$ denotes the set of agents, $AM_{i}$ is the management agent, $AS_{i}$ is one of the *N* service agents maintained by $AM_{i}$ in its domain and *N* is a dynamic constant, which is the number of entities.
- $I=\{i_{0},i_{1},\ldots i_{n}\}$ denotes an *n* step workflow.
- $\Omega^{t}=\times_{i}\omega_{i}$ denotes the finite constraint set.
- $S=\{s_{0},s_{1},\ldots s_{n}\}$ denotes the finite set of services existing in the system.
- $V\left(S^{\prime }|I,R\right)=\sum_{r_{i}\in R_{i}}u_{i}\left(s_{i},r_{i}\right)$ denotes the utility reward value obtained from taking work procedure *I* and $S^{\prime }$ is the actual obtained service set.

Time limitation $T_{i}$ is in terms of the maximum value of the whole service request $R_{i}$, and for each $r_{i}$, it has time limitation $t_{i}$. In each procedure, MA checks and evaluates all of the candidate services, selects the best-matched ones for the current needs while rejecting the others and then returns them to the application. The scheduling process is shown in Figure 5. Similar calculations are carried out in the following subsection.

### 5.1. Constraints Annotation and Matching

The semantic supporting functional layer does not know the upper applications’ needs and constraints. Therefore, in our design, we defined the constraint class to support the instance of constraint annotation. Although the current auto (semi-)semantic annotation technology has emerged, in this paper, we are more concerned with the effect of the constraint annotation and its follow-up, thereby making the annotated technology not our focus here.

In a semantic annotation processing, the annotation can improve the semantic description capability of a service, namely a service can be simultaneously marked and described by its concepts and constraints. In this paper, we built a semantic conceptual service and constraint description graph $G\left(s\right)=\left\{C_{s},C_{t},C_{o}\right\}$. $C_{s}$ denotes the main concepts that correspond to the basic characteristics of the service. $C_{t}$ denotes the constraint predicates, which are used to identify the properties or characteristics of the service specified. $C_{o}$ represents the service object, which can be represented by any concept or text. Similarly, a service in the system can be denoted by G(s), and a service request can also can be denoted by G(r). Therefore, we can define three major constraint predicates:

- isPropertyof, which is used to indicate that concept *A* is an attribute of concept *B*; it shows the conceptual category of *A*, and an instance of *A* can meet part of the requirement of the instance of *B*.
- CanOperate, which is used to indicate that concept *A* can carry out operations on concept *B*; as the aforementioned design, a service can have or operate resources; hence, here, CanOperate refers to the variety of action verbs.
- isRestrictby, which is to indicate that concept *A* is restricted by concept *B*; *B* can be the instance of time, scope, number, etc.

**Figure 13:**
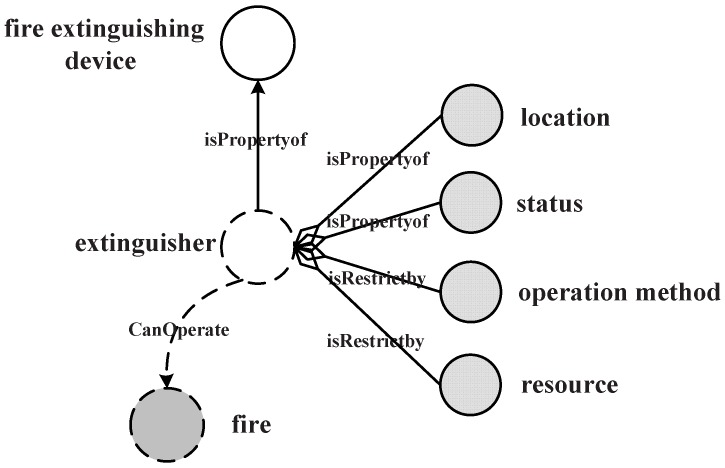
Fire extinguishing service annotation.

As in the above definition, a service can be represented by its basic concepts and constraint annotation triple collections. Figure 13 shows the request query on a 〈fire,extinguishing,device〉 service description fragment. The service returns the extinguisher with location, status and operation method information. The CanOperate information is implied in the mission object, and isRestrictby implied in the constraints. The similarity comparison between each service request and a service is determined by the degree of matching between the constraint description graph and service request graph. For the matching process with the mean values of all similarity comparisons as the *n* matching values, the formula is as follows.

(1) $$\begin{array}{c} sim\left(G\left(s\right),G\left(r\right)\right)=\frac{1}{n}\sum_{i=1}^{n}\left\{sim\left(G_{i}^{s},G_{j}^{r}\right)\right\} \end{array}$$

From Equation (1), $sim\left(G_{i}^{s},G_{j}^{r}\right)=sim\left(op_{r},op_{s}\right)+sim\left(des_{r},des_{s}\right)+sim\left(an_{r},an_{s}\right)$ denotes the similarity between every basic con-triple, corresponding to Operation, Description and Annotation similarities, respectively. The similarity of Operation is denoted as below:

(2) $$\begin{array}{cc} sim(op_{r},op_{s})= & \frac{1}{3n}\sum_{i=1}^{n}\{x_{1}S_{Condition}\left(op_{r}\cdot oCon,op_{s}\cdot oCon\right)\\ & +x_{2}S_{Parameter}\left(op_{r}\cdot oPar,op_{s}\cdot oPar\right)\\ & +x_{3}S_{Method}\left(op_{r}\cdot oDes,op_{s}\cdot oDes\right)\} \end{array}$$

where $x_{i},\;i\in \left\{1,2,3\right\}$ denotes the relative effect category of information. Input and Output are the main features of Parameter. Hence, it can be represented as a tuple: SA(Parameter) = $\langle ⋃\left\{p_{i}\cdot par_{annotation}\right\},\;⋃\left\{p_{i}\cdot par_{annotation}\right\}\rangle$, where $p_{i}$ in the two elements is separately from Input and Output. Description is a common basic description element of both request and service, which can be defined from each component of the natural language representation. The similarity between two concepts can be measured by using the similitude calculation through its collection of keywords. In this paper, we used Dice’s coefficient [28] to calculate the similarity between the keyword set $\left(KEY_{1}\right)$ and $\left(KEY_{2}\right)$ as:

(3) $$\begin{array}{c} sim\left(des_{i},des_{j}\right)=\frac{1}{k}\sum_{i=1}^{k}\frac{2\times |key_{1}|⋂|key_{2}|}{|key_{1}|+|key_{2}|} \end{array}$$

The Annotation exists at the interface, deployment, resource, state, error definition, as well as element type: simple type (xs:SimpleType); complex type (xs:complexType); and attributes (xs:attribute) may have semantic annotations. Therefore, Annotation is an important factor in similarity processing. In this paper, we used Jensen–Shannon information divergence [29] to measure the similarity between two annotations:

(4) $$\begin{array}{c} sim\left(an_{r},an_{s}\right)=\frac{1}{2log_{2}}\sum_{i=1}^{n}\left\{h\left(p_{i},r\right)+h\left(p_{i},s\right)\right\}-h\left(p_{i},r\right)\left(p_{i},s\right) \end{array}$$

where $\left(p_{i},x\right)$ denotes the appearance probability of the index entry *i* in *x*, and h(x)=−xlogx.

As previously mentioned, there may several similar services that can meet the same requirement; hence, for a management agent $MA_{i}$, the service selection problem can be modeled as: a service request consists of *M* minimum service requirement $r_{i}$ and *N* similar service candidates; the similarity relationship between requirements and service candidates is denoted by an M×N matrix. Every element sim(G(s),G(r)) in this matrix represents a vector of matching utility value that is observed by $r_{i}$ on the service candidate $s_{i}$. If $s_{i}$ cannot meet $r_{i}$, sim(G(s),G(r))=0. In every matching process, choose the highest similarity value candidate service as the return.

### 5.2. Service Discovery

Service discovery is used to find the eligible services in the decentralized system. When $MA_{i}$ receives a service request, it will execute the service discovery step by step following the workflow in Figure 5: $MA_{i}$ first checks its local service list; if it can meet the request, it returns the corresponding service(s). If there is any unsatisfied part, $MA_{i}$ will check its neighbors’ services and find out whether any of them has services for the unsatisfied part and then sends the unsatisfied part to the selected neighbors respectively. This process continues until requirements are met or timeout. During this process, there may exist many similar services that can meet one requirement, but are dispersed around the system. In such a case, how to choose the eligible services becomes a big question because that affects the efficiency of task execution greatly.

**Figure 14:**
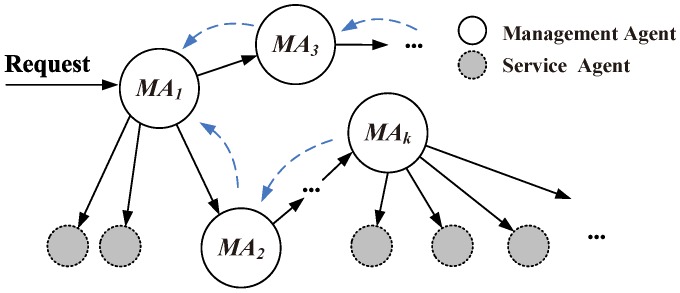
Service discovery via management agents.

As shown in Figure 14, $MA_{i}$ denotes the management agent, and the gray circles denote the service agent maintained by $MA_{i}$. When a service request arrives $MA_{1}$, the arrow lines indicate the direction of request transmission. Therefore, the service discovery problem can be transformed into a transmission problem; the traditional routing approach can be used. According to the service searching procedure definition, we designed a decentralized Semantic-Based Neighbor Discovery (SBND) algorithm and provided a searching strategy to deal with some troublesome points in M2M, such as decentralized and unstable node information and usage scenarios. For a service combination request $R_{i}$, it can be represented as *n* minimum requirement $r_{i}$; each $r_{i}$ is a service request with its lifetime *l* and service quality requirement $\omega_{i}$ as previously mentioned. Therefore, a complete service requirement can be denoted as $\langle R_{i},\Omega_{i},L\rangle$.

To identify the next hop neighbor, $MA_{i}$ maintains an estimation matrix *T* to evaluate its neighbors’ service states. $T_{i}\left[j\right]$ represents $MA_{i}$’s estimation about $MA_{j}$; its current value is determined by the last time the service was accessed. The $T_{i}\left[j\right]$ value is not fixed; it is updated based on the query process between agents. In this case, it can determine the probability of forwarding the service request $r_{i}$ to $MA_{i}$’s neighbors based on the estimation of its neighbors; $P_{i}\left[j\right]$ as $MA_{j}$’s selection probability; $P_{i}\left[j\right]$ is based on $T_{i}\left[j\right]$. threshold expresses the service quality about the request; it is set by the request sender. When the request was being passed, but no agent was able to provide such services needed, this indicates that few management agents in the system can satisfy the threshold. In this case, the threshold value will decrease gradually until the request can be satisfied, on the premise that in the system, there is such a service that can meet the request.

Algorithm 1 illustrates the searching process of agent $MA_{j}$ when it receives $r_{i}$ from its neighbor $MA_{i}$. For each $r_{i}\in R_{i}$, it can use function $matched(MA_{j},r_{i})$ to determine whether the requirement will be satisfied by $MA_{j}$ (Line 4). When a requirement $r_{i}$ is kept, $MA_{j}$ will send a receipt to $r_{i}.path\left[0\right]$, which refers to $R_{i}$’s sender (Line 5). If $MA_{j}$ is unable to satisfy $r_{i}$, it will reduce $r_{i}.lifetime$ (Line 7). If $r_{i}.lifetime$ is no more than zero, its lifecycle is over, such that it cannot be forwarded in the network (Lines 8–9). If $MA_{j}$ decides to pass $r_{i}$, it will add itself to $r_{i}.path$ (Line 11). For all of the neighbors of $MA_{j}$, since they cannot satisfy $r_{i}$, it can reason that the current searching direction in this spread area is risky, so this searching should be terminated, and the searching process should return to the former agent $MA_{i}$ to find the new neighbor with better evaluation (Lines 12–20). As for the previous node of agent $MA_{j}$, since it cannot satisfy $r_{i}.threshold$, this means that the former sender overestimated $MA_{j}$’s service quality; now it needs to bring down the value. For all of the other agents that are within $r_{i}.path$ and that are neighbors of agent $MA_{j}$, it has to detect $r_{i}.threshold$ when $r_{i}$ transmits to this neighbor. However, in the transmission process, $r_{i}.threshold$ has reduced by every time passing through an agent. As a consequence, there is a correction between the $r_{i}.threshold$ value at that time and that of now. The value of cor is related to the numbers of agents that $r_{i}$ has passed through and the step length that $r_{i}.threshold$ has reduced by when passing through every hop. The value is as follows: $cor=\alpha \times [|r_{i}.path|-|loc\left(MA_{j},r_{i}.path\right)|]$; function $loc(MA_{j},r_{i}.path)$ returns the agents sequence in $r_{i}.path$ (starts from zero); *α* is the step length (Lines 13–18). After the $T_{i}\left[j\right]$ vector has been adjusted, the next process is to consider how to update the forward probability of agent $MA_{j}$. Our approach is to make full use of the $T_{i}\left[j\right]$ vector; that is, to normalize the value of $MA_{j}$ forwarding $r_{i}$ to its neighbors, and the larger $T_{i}\left[j\right]$ is, the greater $P_{MA_{j}}$ is. Then, it can judge whether the value of $r_{i}.threshold$ to minimize; if not, then it reduces the value of it. Then, $MA_{j}$ will choose the best neighbor to pass $r_{i}$ according to $P_{j}$ (Lines 14–15).

**Algorithm 1** Searching process of the SBND algorithm.1:**while** true **do**2: **for** all $r_{i}\in R_{i}$ **do**3:  **repeat**4:   **if** $matched(MA_{j},r_{i})$ **then**5:    send receipt to $r_{i}.path\left[\right]$;6:   **else**7:    $r_{i}.l$-=1;8:    **if** $r_{i}.l\leq 0$ **then**9:      kill($r_{i}$) and return unsatisfied message ℵ to $MA_{i}$;10:    **else**11:      append(self, $r_{i}.path$);12:      **for** all $MA_{k}\in \left[r_{i}.path\cup neighbor\left(MA_{j}\right)\right]$ **do**13:       **for** all $MA_{k}\in neighbor\left(MA_{j}\right)]$ **do**14:        **if** $unmatched(\omega_{k},r_{i})$ **then**15:          update $P_{j}\left[k\right]$←$r_{i}.threshold$-=cor;16:        **end** **if**17:       **end for**18:       neighbor $MA_{k}$← choose $argmax(P_{j}\left[k\right])$;19:       **if** $unmatched(\omega_{k},r_{i})$ **then**20:        return unsatisfied message ℵ to $MA_{i}$;21:       **end** **if**22:      **end** **for**23:    **end** **if**24:   **end** **if**25:  **until** $R_{i}$ is satisfied; 26: **end** **for**27: return $S^{\prime }$; 28:**end** **while**


### 5.3. Service Combination Construction

In this subsection, we introduce the service combination approach in detail. In the middleware, each management agent maintains a table to record the combinational service list. An example is presented in Table 4 with the parameters and the activities’ execution information.

For each management agent, it can determine the path of performing tasks and retransmitting information requested according to its combination table. With all of the services done, an integrated combinational service will be formed in terms of accomplished results. Therefore, the whole service combination contains two processes: constructing combination tables and executing the services’ combination. The following introduces each concept in the combination table.

- Initial agent: The original agent starts the services’ combination. The initial agent is responsible for the initialization task of the workflow of the combinational service, such as constructing the global data table, distributing the combinational activity ID, and so on.
- Combinational activity ID: During the process of the combinational task, each activity will be given an ID.
- Target: the target of the executing service requested.
- Prior agent: to record the agent executing the last services’ combination.
- Inferior agent: to record the agent executing the next services’ combination.
- Available agent: to record the agent providing the executing service.

#### 5.3.1. Combination Table Construction

The original form of the combination table is empty; it gradually formed in the process of the execution and discovery of the service over many times. The process contains two stages: dynamic generation and information exchange. When a request comes, the management agent compiles its combination table through the execution of the local services and forwards the request information. Service requests arrive randomly, and during each exchange process, a management agent can obtain more service combination information for its neighbors. The dynamic generation of the combination table will be discussed in the following:

- When receiving a request, the management agent first checks its local records. If there are any, return the services and renew the combination activity ID, the prior agent and the computing assessment of these services. After the current request is completed, the agent continues executing the next request and works as above, and them modifies the prior agent in the table as the current one. The system performs this loop until there is no relevant item in the table or timeout.
- If there is an unsatisfied part in the table, the management agent will search in its local neighbors’ service list. If found, it returns the services and and updates the combination table. Otherwise, the agent would send request information to its neighbors. The neighbor agent needs to renew the combination activity in the previous table of the prior agent. The inferior agent is the most suitable neighbor via computing.
- When the unsatisfied combination table arrives at the new agent, this agent would repeat the first and second steps until the timeout or the number of hops is exhausted.
- In this procedure, if an agent always does not satisfy a certain request type, this agent will be reduced in priority when the same type of request comes, until no selection. If an agent always satisfies a certain request type, its priority will be promoted.

Similar neighbor selection is an important step for service scheduling; in $MA_{i}$’s neighbor list, there may exist *k* neighbors that provide similar services; hence, sending the unsatisfied part $R^{\prime }$ to which neighbor needs to be identified, since different neighbors will decrease the scheduling execution efficiency. Agents in the propagation path are unable to execute requirements, which causes many problems, such as taking too much time and invalid returns. Furthermore, this paper takes the agent’s network degree into consideration. According to related research [30], a higher network degree agent has better data collection. In this way, we redefine the agent’s evaluation function on the basis of the original function.

(5) $$\begin{array}{c} V\left(S^{\prime }|I,R\right)=\sum_{MA_{j}\in \Lambda_{i}^{\prime }}\sum_{r_{i}\in R_{i}^{\prime },i_{j}\in I}P\left(MA_{j}|\Lambda_{i}^{\prime },R^{\prime }\right)\cdot V\left(s^{\prime }|i_{j},r\right) \end{array}$$

In this formula, $\Lambda_{i}^{\prime }$ means the set of agents with higher network degrees and $R^{\prime }$ is the unsatisfied part.

**Figure 15:**
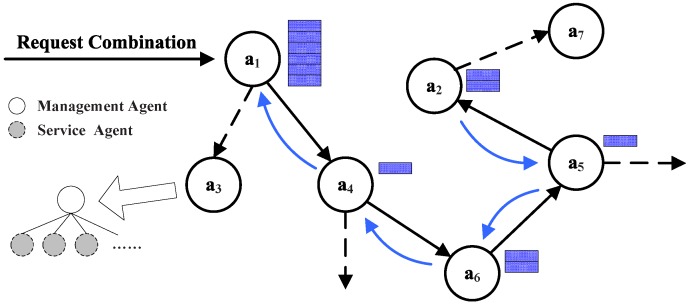
Services combination diagram.

With the execution of services and the formation of the combination table, the services’ combination is also in synchronous execution. It includes two parts: recording the participant and its service and forming the path of services’ combination. As shown in Figure 15, in the initialization, the participant list will be constructed and initiated, for the same services that multiple agents participate in; the chosen agent would be the one with the maximum assessment of service. One agent is likely to be discovered in a sequence more than once under the condition that this agent can provide different kinds of service. Moreover, because the same service can appear in a combination of services many times, a repeating service can be discriminated by combination ID in a participant sequence. Here is given the definition of the participant sequence made of a triad: agent, combination activity ID and assessment. For example, the sequence in Figure 15 shows that agents $a_{4}$ and $a_{6}$ can provide their own services.

Constructing the combination path: The prior agent in the combination table is employed for combining services to constitute the combination path. The agent gets involved in the executing process together with the combination table. When the final task has been executed successfully, the time is up or the limitations of jumps have been used up; the last agent of the execution starts to go back to its prior agent and constructs the combination path. This path is shown in Figure 15 as arrows.

#### 5.3.2. Service Combination Process

Service combination is not the simple superposition of multiple services, but rather, a composition of different services according to the characteristics of the interaction. Therefore, it should be emphasized that all service combination processes need to be established based on the specific domain knowledge, whether it is expert defined or based on context awareness. When $MA_{i}$ receives a request, it computes all of the semantic relations between the relevant services for the request. The service combination can be described as a directed acyclic graph, G=(V,E), where:

- $V=S_{i}⋃A$ is the set of vertices of the graph, where *S* is the set of services, and $A\in R_{i}$ is the set of annotations from services requested (inputs and outputs).
- *E* is the set of edges in the graph indicating the connections between the services.

**Figure 16:**
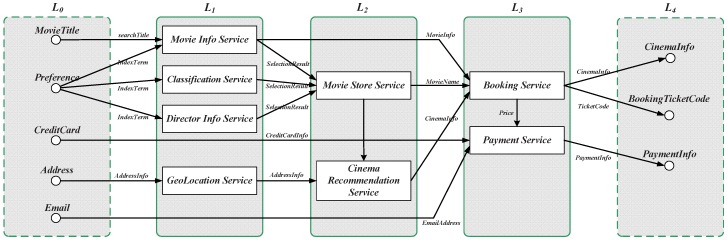
Services’ Combination example of booking movies.

This graph contains all of the candidate services that could directly be invoked by $MA_{i}$. The graph is divided into *K* layers, and two special layers, namely $L_{0}$ and $L_{K+1}$, contain the dummy services as the input request $IR_{i}$ and output result $OS_{i}$, respectively. An example of an online movie booking service is $L_{0}$: $IR_{i}=\left\{MovieTitle,Preference,CreditCard,Address,Email\right\}$ and $L_{4}$: $OS_{i}=\left\{CinemaInfo,BookingTicketCode,PaymentInfo\right\}$, as shown in Figure 16. As previously mentioned, the first step of the service composition construction is to calculate the relevance between candidate services and requests. These services can be easily calculated layer by layer, using the matching mechanism in the previous sections. The algorithm selects from the set of all available services in each layer, by using the relevant calculation Equation (1), for each candidate service, and performs a match between the input annotations and the services by using the matching method defined in Section 5.1. All of the candidate services are calculated from those unmatched by using the similarity calculation method defined in Section 5.2. For example, the first eligible service set for the request shown in Figure 16 is the services in the layer $L_{1}$, which correspond with the inputs in $L_{0}$. The second eligible service set is those services in the layer $L_{2}$, whose inputs are fully matched with the previous layers’ outputs, and so on. The complexity analysis of service composition construction is: $O(l\cdot m\cdot n\cdot \frac{w}{k})$. The first part corresponds with the complexity of the matching process, which costs *l* times; the second part corresponds with the complexity of the similarity calculation process, which costs *m* times; whereas the third part corresponds with the complexity of the looping accessibility check for each candidate service, which costs *m* times. It can expected that there is a subset of the candidate services *w* invoked from all of the candidate services *k*. Thus, $\frac{w}{k}$ is a reduction factor that depends on the number of candidate services.

## 6. Simulation and Results

To manifest the feasibility and efficiency of our design, we designed a smart city fire hazard response scenario; we built a hypothetical urban area stochastic fire hazard incident in Unity3D. The simulation parameters in Himoto’s work [31] are maintained for our work. The parameters and properties relating to the burning of the building are shown in Table 5. The fire model and the effect data are adopted from the Wildland urban interface Fire Dynamics Simulator (WFDS) simulation [32]. The communication model and protocols are also from our previous work [33].

**Figure 17:**
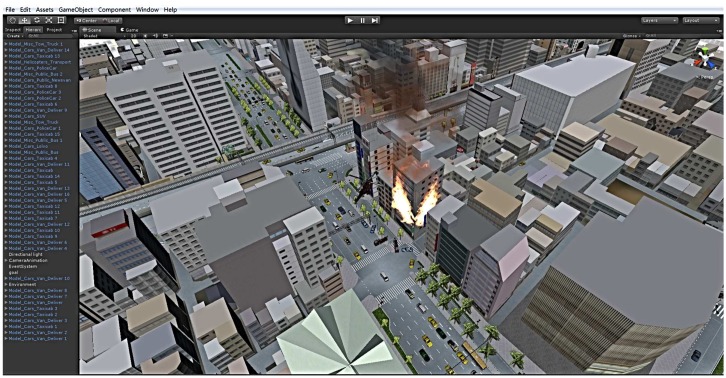
Scenario of fire hazard response.

In this simulation scenario, the police station, fire company and hospital are scattered around the urban area; the corresponding police cars, fire engines and ambulances can be invoked, and their corresponding service agents are built in the middleware. In addition, a surveillance agent is defined to monitor the fire hazard; all of these social entities are used to achieve the provision of various services through their respective SAs. The middleware maintains three types of management agent: the fireman MA is in charge of the fire engine SAs’ management; the security MA manages the police cars SAs; the rescue MA manages the ambulance SAs. In addition, traffic, meteorological and some other useful information can be obtained through the traditional web service interface; the urban vehicles are generated and controlled by the platform. However, since this aspect is not the focus of this paper, we provide no further details about that. The fire hazard response is as shown in Figure 17; according to the calculations and scheduling, finally, three police cars, two fire engines and one ambulance were summoned to the fire scene from the near departments.

Different searching approaches will not have much impact on small-scale system performance, but in a large-scale system, they will. In the algorithms’ comparison, we compared our SBND algorithm with two other approaches: multi-cast [34] and random. In the middleware, each management agent randomly maintains 1–10 different types of service agents. Each time, the management agents construct a small-world network; this ensures the network connectivity. Hence, in the following experiments, multi-cast applied the typical shortest path tree approach; the root of the tree is the request agent of the multi-cast; the searching over the network formed the branch. In a random approach, each time from the request agent, it randomly selected a neighbor to check the requirement. All of the simulation results are averaged over 100 runs; the experimental results are as follows.

**Figure 18:**
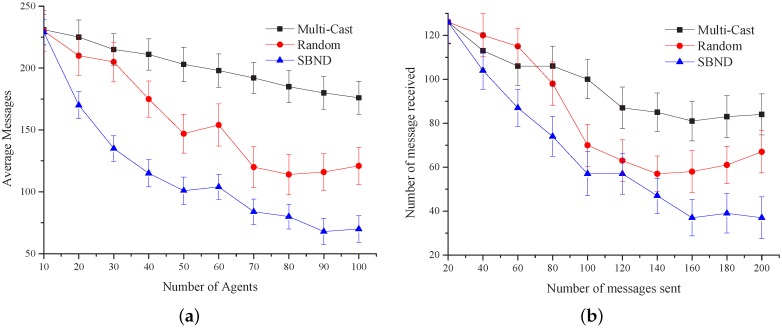
Experimental results’ comparison over three approaches. (**a**) Average messages received by agents; (**b**) relationship between sent and received.

Figure 18a shows the average messages that agents received with the three algorithms. The simulation results reveal that the average number of received messages reduces when the agents’ number scales up and is finally stable until convergence is achieved. The average number of messages is about 20% less than the messages received in the random algorithm and is about 60% less than the messages received in the multi-cast algorithm. This means that the algorithm we presented in this paper can decrease the number of messages transmitted in the network to some extent. We can also see that the random algorithm fluctuates largely, while our algorithm is relatively smooth.

Figure 18b shows the relationship between the numbers of messages sent and received. The *X*-axis represents the average number of agent messages sent, and the *Y*-axis represents the average number of agent messages received. After statistical calculations, we found that the average number of messages received in the random algorithm is 14% more than our algorithm. As to the multi-cast algorithm, it is 45% more, which indicates that our algorithm performs better than the two.

**Figure 19:**
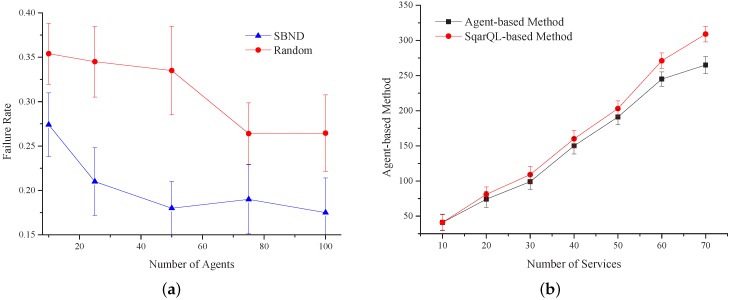
Experimental results’ comparison. (**a**) Relationship between sent and received; (**b**) failure rate of two mechanisms.

Figure 19a shows the comparison of the failure rate under two different service transmission mechanisms based on the designed middleware system. The figure illustrates the failure probability of the requirement when executing the combined tasks. We compared our algorithm with the random algorithm. This figure shows that the convergence rate of our algorithm is faster than the random algorithm. When the number of agents is 40, the failure rate of our algorithm decreases quickly, and then, it maintains a constant value at 0.18. However, in the random algorithm, the failure rate will be stable after the agent number reaches 60, and the failure rate will keep at about 0.23. From the above analysis, the convergence rate of our algorithm is faster, while its failure rate is 0.05 less than the failure rate in the random algorithm.

The service executing time is a very important evaluation index. In this regard, we used the algorithm implemented by the SqarQL-based method to compare with our agent-based design. Figure 19b shows that when the number of services is less than 40, the performance of the SqarQL-based method basically coincides with that of our method and sometimes even better. When the number of services increases, our agent-based method outperforms the SqarQL-based method in terms of executing time. When the agents are initialized, they do not have much information about their neighbors. As the services executed increases, the exchange of the combination among agents increases, as well, and the information agents have about their neighbors gets more accurate. When executing tasks, agents can choose a more reasonable neighbor to forward the demands. Thus, the accuracy of executing tasks can be improved; meanwhile, the executing time can be saved.

## 7. Conclusions and Future Works

In this paper, we proposed an agent-based, service-oriented middleware towards semantic service enablement in M2M application and a prototype implementation of this middleware. This middleware provides a semantic service representation model to support inter-operability between heterogeneous M2M services. It further enhances the specification of semantic services in a decentralized M2M environment using semantic annotation in sensed data. Furthermore, it was realized that semantically-organized service descriptions can effectively improve the efficiency of querying and locating services. As part of the middleware design, we present an efficient semantic service discovery and matching approach for the service combination process, which calculates the semantic similarity between services and compares services with respect to their suitability for a specific service request, so that selection can be made among them. Anchored on the integration of service discovery and matchmaking within the composition process, we also gave a theoretical analysis of service composition in terms of its dependency with service discovery. Finally, the comprehensive experimental results show the effectiveness and feasibility of our design.

However, with standardization processes mostly taking long times to complete, the existing M2M applications are still faced with several significant barriers, such as current systems have strong independence, heterogeneity and difficulties on code reuse. These issues will be more complex in smart cities over the next decade. In addition, although some current research provides a registration-based service discovery and response mechanism, services are still scattered in systems. The formation and discovery of the complex combination of services are still difficult jobs for the future.

## Figures and Tables

**Table 1:** Current typical M2M platforms.

Platform	Protocol	Interface	Resource Share	Data Analysis	Characteristics
ThingSpeak	HTTP	RESTful	△	△	Real-time data, Ruby, Integrated in ioBridge.
AllJoyn	HTTP	RESTful	△	△	RTOS, Arduino, Linux, Android, cloud services.
Nimbits	HTTP	RESTful	△	△	Event Driven, Rule based, Google App Engine, Amazon EC2, Java.
Bugswarm	HTTP	RESTful	△		Tag and Rule, Linux, 3G, WiFi.
RIOT	CoAP	RESTful	△		6LoWPAN, IPv6, RPL , support embedded devices, C, C++.
Nitrogen	HTTP	RESTful		△	ZigBee, WiFi, support embedded devices, home environment.
SensorCloud	HTTPS	RESTful		△	MathEngine, MicroStrain sensors.
ThingWorx	HTTP			△	Enterprise oriented, cloud service.
Axeda	HTTPS	RESTful		△	Axeda Wireless Protocol, cloud service.
openAlerts	HTTP/CoAP	RESTful			ZigBee, Linux/BSD , e-mail or text message alerts.

CoAP: Constrained Application Protocol. RPL: IPv6 Routing Protocol for Low-Power and Lossy Networks. BSD: Berkeley Software Distribution for Unix.

**Table 2:** Execution states of the management agent. Cons., Constraint; Req., Requirement; Eval., Evaluate.

States	Sub-Goal	Plans
Request	Get appropriate services	Cons. annotation, Req. decomposition
Match	Find better match	Query, generate combination
Execution	Monitoring	Scheduling, Eval. matching
Pause	Recover	Performance evaluation, update execution
Succeed	Secede	Return outcome
Cancel	Cancel request	Notify cancellation

**Table 3:** Execution states of the service agent.

States	Sub-Goal	Plans
Maintain	Update	Registration semantic annotation
Pause	Recover pause	Performance Eval. renewal
Cancel	Cancel registration	Notify cancellation

**Table 4:** A instance of the combination table.

Initial Agent	Combination Activity ID	Target	Prior Agent	Inferior Agent	Available Agent	State	Assessment
A1	006	Temperature	A5	A2	A6	Active	0.85

**Table 5:** Hypothetical urban and building parameters.

City Area Size	10 × 10 km^2^
Number of buildings	500 (dimensionless)
Side length of a building	8–100 m
Building separation	5–150 m
Wind velocity	2.5–10.0 m/s
Thermal conductivity	0.15 × 10^−3^ kW/mk
Wall density	500 kg/m^3^
Heat capacity	1.8 kJ/kg·K
Burn-through time	600 s
Growth rate factor	1.0 × 10^−3^ m^2^/s^2^
